# Influenza Epidemic Trend Surveillance and Prediction Based on Search Engine Data: Deep Learning Model Study

**DOI:** 10.2196/45085

**Published:** 2023-10-17

**Authors:** Liuyang Yang, Ting Zhang, Xuan Han, Jiao Yang, Yanxia Sun, Libing Ma, Jialong Chen, Yanming Li, Shengjie Lai, Wei Li, Luzhao Feng, Weizhong Yang

**Affiliations:** 1 Department of Management Science and Information System Faculty of Management and Economics Kunming University of Science and Technology Kunming China; 2 School of Population Medicine and Public Health Chinese Academy of Medical Sciences & Peking Union Medical College Beijing China; 3 Department of Respiratory and Critical Care Medicine Affiliated Hospital of Guilin Medical University Guilin China; 4 Department of Respiratory and Critical Care Medicine Bejing Hospital Beijing China; 5 WorldPop School of Geography and Environmental Science University of Southampton Southampton United Kingdom; 6 The First People’s Hospital of Yunnan Province Affiliated Hospital of Kunming University of Science and Technology Kunming China

**Keywords:** early warning, epidemic intelligence, infectious disease, influenza-like illness, surveillance

## Abstract

**Background:**

Influenza outbreaks pose a significant threat to global public health. Traditional surveillance systems and simple algorithms often struggle to predict influenza outbreaks in an accurate and timely manner. Big data and modern technology have offered new modalities for disease surveillance and prediction. Influenza-like illness can serve as a valuable surveillance tool for emerging respiratory infectious diseases like influenza and COVID-19, especially when reported case data may not fully reflect the actual epidemic curve.

**Objective:**

This study aimed to develop a predictive model for influenza outbreaks by combining Baidu search query data with traditional virological surveillance data. The goal was to improve early detection and preparedness for influenza outbreaks in both northern and southern China, providing evidence for supplementing modern intelligence epidemic surveillance methods.

**Methods:**

We collected virological data from the National Influenza Surveillance Network and Baidu search query data from January 2011 to July 2018, totaling 3,691,865 and 1,563,361 respective samples. Relevant search terms related to influenza were identified and analyzed for their correlation with influenza-positive rates using Pearson correlation analysis. A distributed lag nonlinear model was used to assess the lag correlation of the search terms with influenza activity. Subsequently, a predictive model based on the gated recurrent unit and multiple attention mechanisms was developed to forecast the influenza-positive trend.

**Results:**

This study revealed a high correlation between specific Baidu search terms and influenza-positive rates in both northern and southern China, except for 1 term. The search terms were categorized into 4 groups: essential facts on influenza, influenza symptoms, influenza treatment and medicine, and influenza prevention, all of which showed correlation with the influenza-positive rate. The influenza prevention and influenza symptom groups had a lag correlation of 1.4-3.2 and 5.0-8.0 days, respectively. The Baidu search terms could help predict the influenza-positive rate 14-22 days in advance in southern China but interfered with influenza surveillance in northern China.

**Conclusions:**

Complementing traditional disease surveillance systems with information from web-based data sources can aid in detecting warning signs of influenza outbreaks earlier. However, supplementation of modern surveillance with search engine information should be approached cautiously. This approach provides valuable insights for digital epidemiology and has the potential for broader application in respiratory infectious disease surveillance. Further research should explore the optimization and customization of search terms for different regions and languages to improve the accuracy of influenza prediction models.

## Introduction

The seasonal influenza epidemic poses a persistent and severe threat to global public health [1]. Over the past century, 4 influenza pandemics have occurred at intervals of 10-50 years [2], resulting in a staggering 0.5-50 million deaths [3-5]. The greatest threat is the uncertainty surrounding the time, location, and causative pathogen of these emerging and reemerging infectious diseases. Because the influenza A virus has a wide range of hosts and can infect wild birds, poultry, pigs, horses, humans, and other animals, elimination of the influenza virus is unrealistic from the perspective of the natural ecological distribution of the virus. Respiratory infectious diseases have no borders; therefore, the surveillance of each country or region is significant to other countries and global health.

The rapid spread of these infectious diseases not only endangers the public health of the population but also leads to a surge in medical demand, straining the health care infrastructure. Effective disease prevention and control measures can help significantly reduce or prevent further damage to population health, society, and the economy. Hence, early detection of infectious disease outbreaks is essential for specific regions affected by the outbreak as well as for the development of global seasonal influenza prevention and control.

The growing consensus that traditional surveillance systems and early warning data are inadequate for tackling current pandemics indicates the emergence of a complex public health concern. Conventional syndromic surveillance relying on case reporting suffers from delays due to tendencies to seek medical attention, laboratory confirmation, and reporting procedures, hindering the timely detection of outbreaks of influenza [6,7]. As a result, studies have explored alternative data sources for early outbreak detection, such as analyzing over-the-counter medication sales and school absenteeism data [8,9]. The World Health Organization has developed a new model for the surveillance of emerging threats, referred to as “pandemic and epidemic intelligence,” which builds upon a range of traditional [10] and nontraditional approaches [11]. The application of the “detection of signals of public health events from unstructured textual web-based information, including social media channels” [11] was included as a modern approach to pathogen surveillance.

Modern pathogen surveillance offers a broader application scope beyond national boundaries with robust surveillance systems and can be extended to developing countries with limited surveillance capabilities. As of the second quarter of 2022, Baidu, the most widely used search engine in China, had more than 600 million active users worldwide. The first effort to use internet search engines for biosurveillance was reported in 2008 when the Google Flu Trend (GFT) was launched, which displayed real-time updates of flu-related events in the United States 1 week before the publication of government-led flu reports [12]. However, the flu predictions of GFT significantly underestimated the H1N1 pandemic in 2009 and failed to produce an accurate flu trend during the 2012-2013 flu season in the United States. After several attempts, the role of big data in influenza surveillance was doubted, especially in developed countries [13]. However, big data have been crucial during the COVID-19 pandemic, especially for active tracking in China. This significant increase in the use of big data has alerted researchers and the tech industry of its usefulness in preventing and controlling infectious diseases.

Existing studies have largely been based on a single city or a crude national analysis based on monthly data. However, whether modern data are available in both north and south climates remains to be explored. Therefore, we developed a predictive model for influenza supplied by Baidu search query data to combine traditional surveillance with modern big data. This study aimed to detect new infectious disease outbreaks earlier and better prepare for such outbreaks.

## Methods

### Ethics Approval

In accordance with ethical guidelines, this research received approval from the Ethics Review Committee of Peking Union Medical College Hospital, Chinese Academy of Medical Sciences (CAMS&PUMC-IEC-2021-032).

### Source and Collection of Influenza Virological Data and Baidu Query Data

The virological data were retrieved from our previously published study [14], which used data from the National Influenza Surveillance Network. The Chinese Center for Disease Prevention and Control monitors 554 sentinel hospitals and 407 network laboratories throughout each province of mainland China. From these centers, a total of 3,691,865 samples were detected positive for influenza from January 1, 2011, to July 31, 2018.

The Baidu query data were retrieved from the public Baidu index website [15], which covered the 31 provinces of mainland China and were classified as personal computer port or mobile port source data. The Baidu search terms involved a total of 1,563,361 samples from January 1, 2011, to July 31, 2018.

### Baidu Search Term Selection

**Figure 1:**
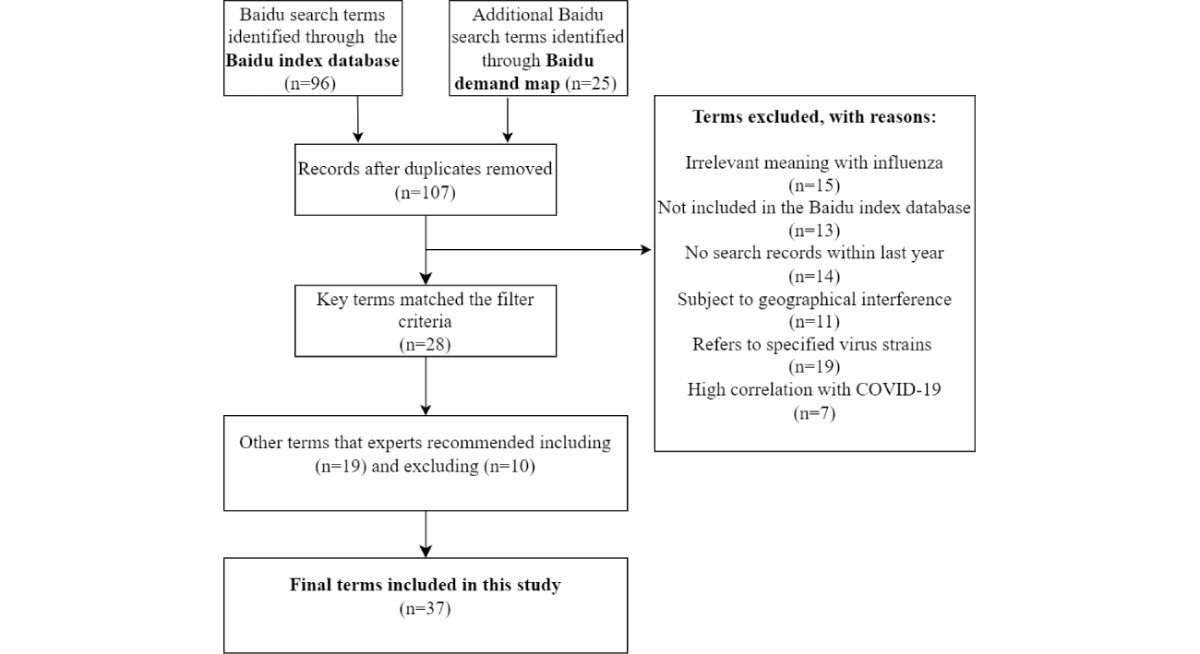
Baidu search terms selection procedure (details in Table S1 in Multimedia Appendix 1).

Potentially relevant search terms were determined using the procedure illustrated in Figure 1. Primary indicator terms to identify more related queries about seasonal influenza were found in 2 data sources. The first was an open-access Chinese website [16], a free platform that provides internet keyword mining of the Baidu search engine in mainland China. The second was the Baidu index website. Overall, 107 keywords were included in the primary database. After excluding non–flu-related news and movies and words related to the pandemic, avian influenza, COVID-19, and those specific to certain regions, 37 words were retained.

### Correlation Analysis Between Influenza-Positive Rate and Baidu Search Terms

Pearson correlation coefficients (PCCs) between the included Baidu search terms and the influenza-positive rate were calculated. First, we merged the synonyms into 1 combined search term. Subsequently, the PCC between each Baidu search term and the influenza-positive rate was calculated using equation 1, and statistical significance was set as *P*<.05. According to the intensity of the correlation, we classified the correlation as high (PPC>0.6-0.7), medium (PPC 0.4-0.6), and low (PPC<0.4).



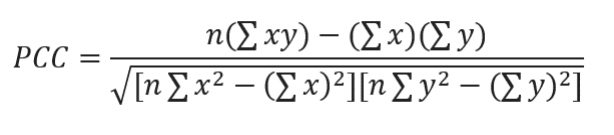



Here, *PCC* refers to the Pearson correlation coefficient, *x* refers to the values of the Baidu search index data, *y* refers to the values of the influenza-positive rate data, and *n* refers to the total number of values.

### Distributed Lag Nonlinear Model Lag Correlation Analysis

The lag correlation of the Baidu search index was analyzed using a distributed lag nonlinear model (DLNM), that is, a regression model in a nonlinear process [17], as described in equation 2.



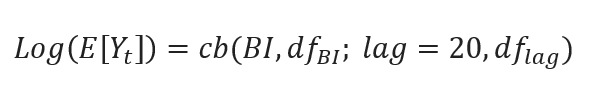



*E[Yt]* refers to the time series of the daily influenza-positive rate in northern and southern China, and *cb(BI)* refers to the cross-basis matrix of the time series of daily Baidu search terms, respectively. The natural cubic spline function was adopted in all the element spaces, and a lag period of 1-20 days was selected when establishing a cross-basis matrix. The quasi-Poisson function was used as the connection function in the model to control the overdispersion effect. Relative risks were used to assess the lag-response relationships of single-day effects.

Before DLNM analysis, all Baidu search terms were divided into 4 categories: general influenza information, influenza prevention, influenza treatment and medicine, and influenza symptoms. The DLNM was used using R Studio and R software (version 4.1.2; R Core Team), specifically the *dlnm* [18] package.

### Influenza-Positive Rate Prediction Model

**Figure 2:**
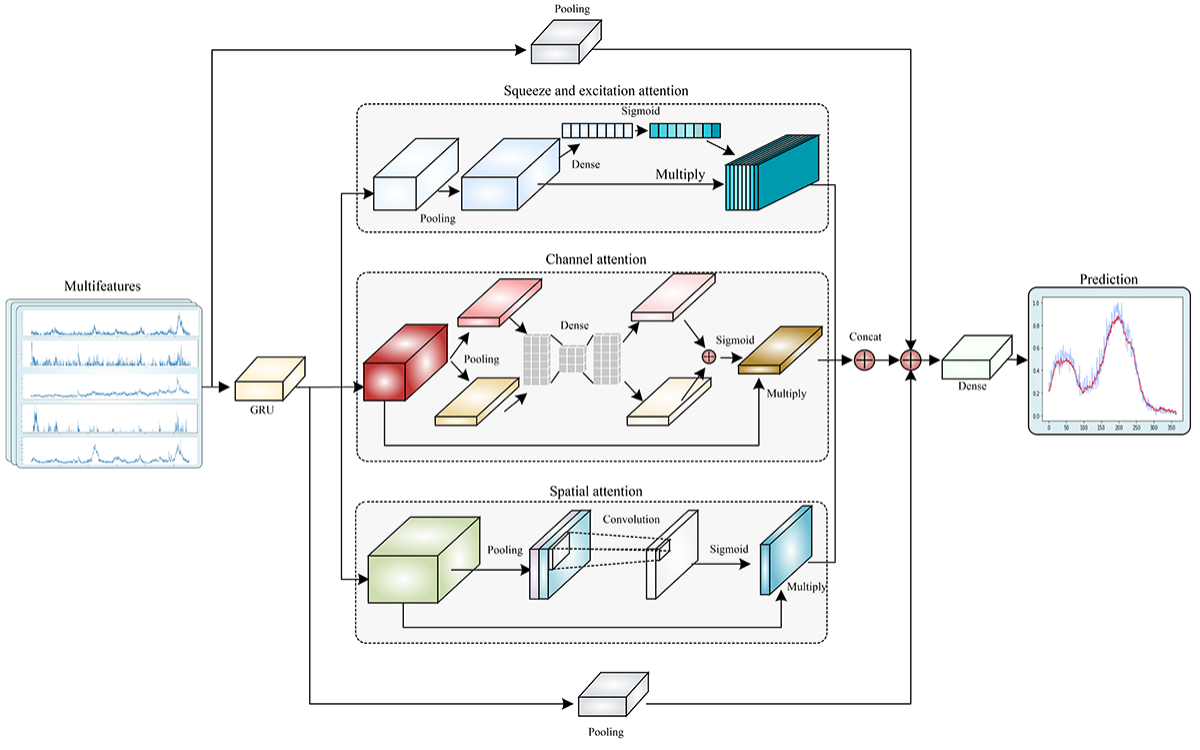
Structure of the gated recurrent unit and multiple attention mechanisms model based on the gated recurrent unit, squeeze and excitation attention, channel attention, and spatial attention. The schematic diagram represents different categories of indexed Baidu data input to the model, including essential facts on influenza, symptoms, treatment and medicine, and prevention. The curves of 5 independent variables are displayed: viral influenza, seasonal influenza, influenza virus, influenza prevention knowledge, and symptoms of influenza.

In this research, we built a prediction model for multifeature time series based on the gated recurrent unit and multiple attention mechanisms model (Figure 2). By fully mining the inherent characteristics of multisource data and establishing the mapping relationship between features and results, the model can complete the task of time series prediction in a scientific and robust manner.

After receiving the multifeature inputs, we connected them to the gated recurrent unit layer, which processes the time series and captures the relationship between the step spacing in a time series. Subsequently, we concurrently used 3 different attention mechanism modules, namely, squeeze and excitation attention [19], channel attention, and spatial attention [20], which not only extract the important information among the different features but also the critical information within the same feature. Moreover, to prevent the disappearance of the gradient, after splicing the results of the different attention modules, we also connected the results with 2 pooling layers for residual connection and finally through the dense layer to output the predicted results.

The training interval was from January 1, 2011, to May 31, 2017, the validation interval was from June 1, 2017, to August 31, 2017, and the prediction interval was from September 1, 2017, to July 31, 2018. According to the PCCs, the Baidu search terms were classified into 4 levels, namely, correlation (*P*<.05), regular correlation (PCC≥0.4), and strong correlation (PCC≥0.6-0.7). The *R*^2^ and SE were calculated by comparing the predicted and actual data to identify the best model with different correlation levels of Baidu search terms. To identify the influence of different days in advance on the prediction effect, this study distinguished 30 cases where the prediction began 1-30 days in advance. The composite prediction model was constructed using Python (version 3.6; Python Software Foundation).

## Results

### Correlation Analysis Between Baidu Search Terms and Influenza-Positive Rate

Based on the literature and expert consultation, the Baidu search terms we screened out were all related to influenza. Overall, we observed a high correlation between the Baidu search terms and the influenza-positive rate in all databases except the term 胸闷 (“sense of suppression in the chest”). The terms were classified into 4 categories: basic understanding of influenza, symptoms, treatment and medicine, and prevention (Table 1 and Figure S1 in Multimedia Appendix 1). The correlation coefficients differed between northern and southern China.

**Figure 3:**
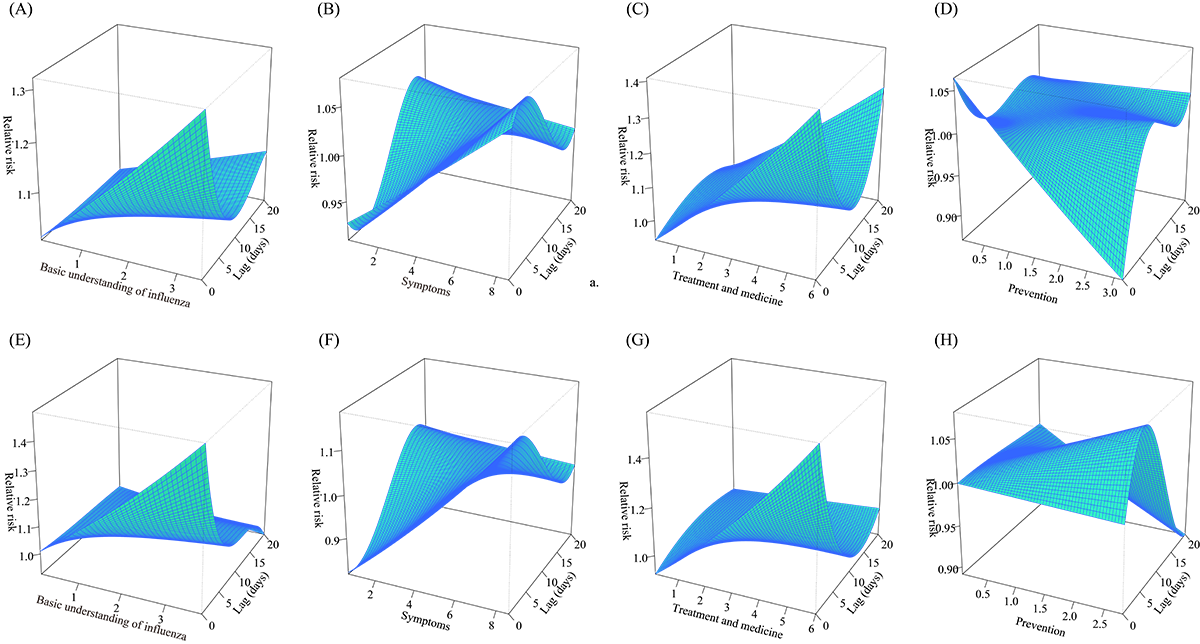
Lag correlation analysis between Baidu search terms and influenza-positive rate. A-D represent northern China. E-H represent southern China. A and E, B and F, C and G, and D and H represent the basic understanding of influenza, symptoms, treatment and medicine, and prevention, respectively.

The lag of relevant days varied according to the search term category. Search terms related to the basic understanding of influenza, treatment, and medicine showed no associated lag. Meanwhile, symptoms-related terms showed a lag correlation with influenza activity of 1.4 days in northern China and 3.2 days in southern China. Prevention showed a lag correlation with influenza activity of 5.0 days in northern China and 8.0 days in southern China. The lag correlation trend of Baidu search indexes in the same category was similar between northern and southern China (Figure 3).

**Table 1:** Pearson correlation analysis between Baidu search terms and seasonal influenza-positive rate in northern and southern China.

Category and terms in Chinese		Terms in English	Northern China					Southern China		
			PCC^a^		*P* value		PCC		*P* value	
**Essential facts on influenza**						<.001				<.001
	流感	influenza	0.48				0.41			
	感冒	common cold	0.40				0.23			
	病毒性流感	viral influenza	0.72				0.61			
	季节性流感	seasonal influenza	0.21				0.29			
	流感病毒	influenza virus	0.55				0.38			
	流感传播途径+流感的传播途径	route of influenza transmission + influenza transmission route	0.26				0.37			
	预防流感知识	influenza prevention knowledge	0.17				0.09			
**Symptoms**										
	发烧	fever	0.40		<.001		0.28		<.001	
	发热	febrile	0.42		<.001		0.09		<.001	
	咳嗽	cough	0.11		<.001		0.21		<.001	
	咽喉痛+嗓子痛	sore throat + pharyngalgia	0.28		<.001		0.19		<.001	
	流涕	runny nose	0.12		<.001		0.08		<.001	
	肺炎	pneumonia	0.28		<.001		0.16		<.001	
	胸闷	sense of suppression in the chest	N/A^b^		.07		N/A		.20	
	流感的症状	symptoms of influenza	0.68		<.001		0.6		<.05	
	打喷嚏	sneeze	0.12		<.001		0.07		<.001	
	乏力	lack of strength	–0.1		<.001		–0.07		<.001	
	肌肉酸痛	muscle soreness	–0.11		<.001		–0.06		.002	
**Treatment and medicine**						<.001				<.001
	流感治疗	influenza treatment	0.68				0.60			
	感冒药	cold medicine	0.3				0.22			
	退烧药	febrifuge	0.39				0.25			
	连花清瘟	Lianhuaqingwen	0.34				0.26			
	流感丸	Liuganwan	0.48				0.26			
	感冒清热+板蓝根+白加黑	Ganmaoqingre + Banlangen + Baijiahei	0.24				0.14			
	奥司他韦+达菲	Oseltamivir + Tamiflu	0.5				0.41			
**Prevention**										
	流感疫苗	influenza vaccine	0.22		<.001		0.31		<.001	
	流感的预防+流感的预防措施+预防流感+怎样预防流感	prevention of influenza + preventive measures of influenza + influenza prevention + how to prevent influenza	0.50		<.001		0.48		<.001	
	流感疫苗副作用	side effects of influenza vaccine	0.04		.04		0.07		<.001	
	流感疫苗有必要打吗	is it necessary to take influenza vaccine	0.1		<.001		0.15		<.001	

^a^PCC: Pearson correlation coefficient.

^b^N/A: not applicable.

### Comparison of the Prediction Effect Within Different PCC Levels of the Baidu Search Terms

Upon comparing the effect of different search terms on the prediction, more supplemental search terms were found to occur in northern China, exhibiting a worse prediction effect. Conversely, the best predictions in southern China were demonstrated by supplementing all relevant search terms (Table 2).

**Table 2:** Supplemented different correlation levels of Baidu search terms on the prediction.

Location and PCC^a^		*P* value	*R* ^2^	Mean squared error	Days when *R*^2^ was >0.8, n	Days when *R*^2^ was >0.9, n
**Northern China**						
	All	<.001	0.775	0.175	8	12
	*r*≥0.4	<.001	0.837	0.013	7	12
	*r*≥0.6	<.001	0.880	0.009	9	18
	*r*≥0.7	<.001	0.891	0.008	14	20
	N/A^b^	N/A	0.895	0.008	14	22
**Southern China**						
	All	<.001	0.893	0.009	11	21
	*r*≥0.4	<.001	0.864	0.011	6	16
	*r*≥0.6	<.001	0.865	0.011	7	15
	N/A	N/A	0.864	0.011	10	18

^a^PCC: Pearson correlation coefficient.

^b^N/A: not applicable. These values represent historical influenza data without Baidu search data involved in the model.

### Testing the Prediction at Different Times in Advance

**Figure 4:**
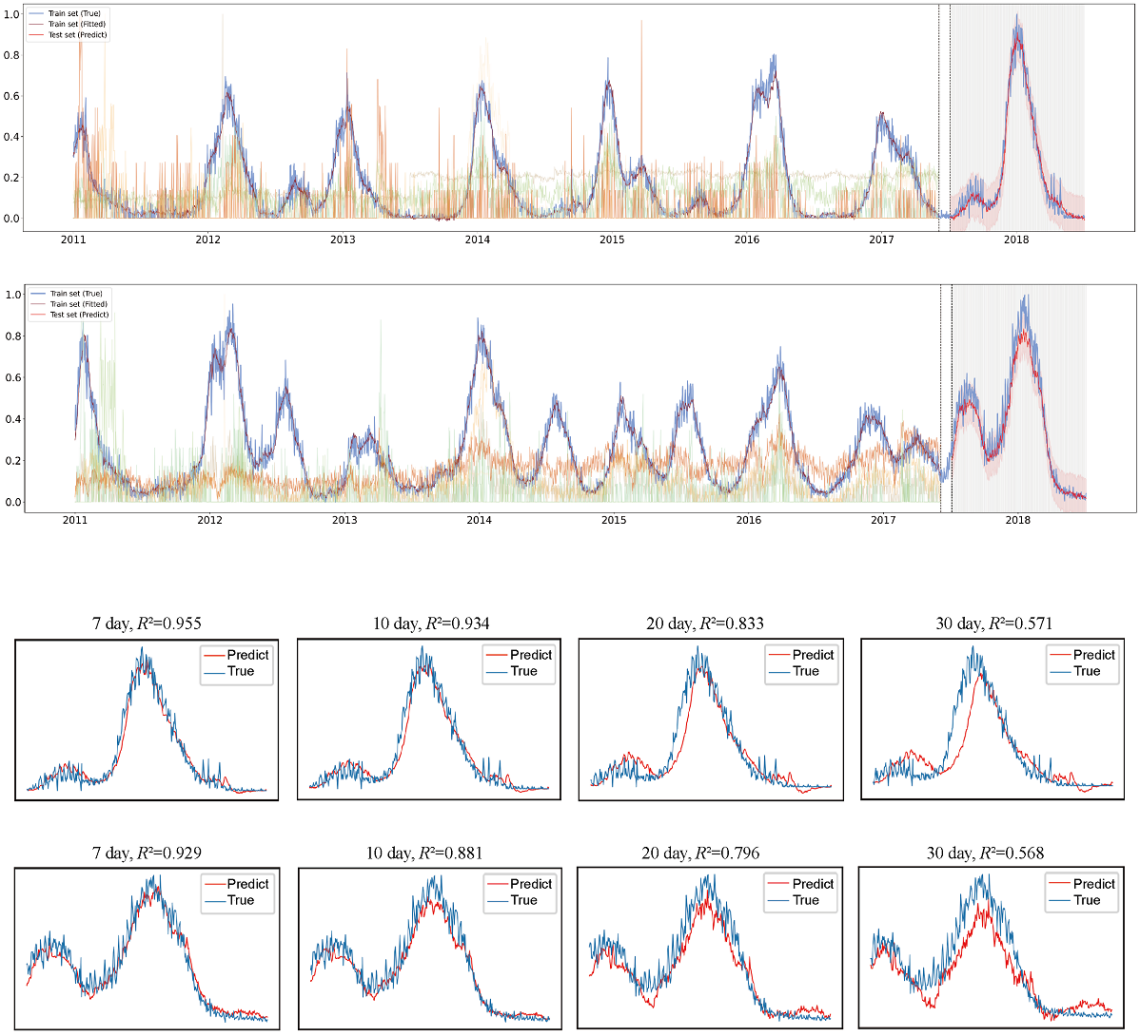
Prediction of influenza-positive rates 1-30 days in advance. A and C-F represent northern China. B and G-J represent southern China. A and B, C and G, D and H, E and I, and F and J show the predicted results 1, 7, 10, 20, and 30 days in advance. Yellow and green lines represent the trend of Baidu search terms.

We chose the optimization model to test the prediction at different times. Northern China showed an *R*^2^>0.9 and 0.8 for the influenza-positive trend prediction 14 days and 22 days in advance, while that in southern China was 11 days and 21 days in advance, respectively (Figure 4). We also predicted the influenza-positive trend in northern and southern China under the Baidu search index data incorporating different correlation coefficients, and the results are shown in Figures S2-S8 in Multimedia Appendix 1.

## Discussion

Predicting seasonal influenza has proven to be a great challenge based on the traditional surveillance system and simple algorithms. Following the application of big data and updated technology, the World Health Organization proposed a contemporary model. This study explored the application of combining Baidu search data with the traditional surveillance system to predict influenza outbreaks. This will not only provide a reference for southern and northern China but also for a modern surveillance approach.

Baidu search term–based surveillance can be applied to seasonal influenza, COVID-19, and other respiratory infectious diseases. Syndromic surveillance can detect outbreaks of disease or adverse health events earlier than traditional forms of public health surveillance [21] and avoid delay in seeking medical attention among patients. We analyzed the correlation between the Baidu search terms and the seasonal influenza-positive rate, and terms related to basic influenza knowledge, symptoms, treatment and medicine, and prevention were found to be highly correlated with the seasonal influenza-positive rate. However, search queries may vary by country and language. A study on GFT found that 26 Dutch terms shared a high correlation with the incidence of influenza-like illness [10]. Regarding the Korean search engine, Daum, a study found 13 combined queries with *r* values ≥0.7 [22]. In Italy, a study found 5 entry terms with high correlation coefficients >0.6 [23]. Therefore, the related research on influenza prediction and the future improvement of influenza surveillance systems can make the above categories of relevant search terms in China as a reference. Cross-references can be made for different countries and language systems; however, individualized analyses should be performed.

Increasing the use of big data is not always beneficial. Not all relevant search terms positively affect the accuracy and timeliness of influenza prediction. Inconsistent with previous research, this study demonstrated that increasing the use of Baidu search terms reduces the accuracy and time before influenza prediction. The same conclusion has been confirmed in GFT-related studies, which indicated that GFT data might not provide reliable surveillance for seasonal or pandemic influenza [24]. This may be because climate and strain, which are important factors affecting influenza, were not considered. Existing studies have shown that the contribution of the Baidu search index to influenza prediction was much lower than that of climate when climate and Baidu search indexes were used to construct a prediction model of influenza [25].

In light of these findings, it is crucial to recognize the broader public health implications of this study. First, while this study used data solely from the Baidu search engine, it is important to acknowledge that Baidu is primarily used in China and may not represent search behavior in other countries. As a result, the generalizability of these research findings to other regions or search engines may be limited. However, the methodology used in this study could be adapted and extended to incorporate data from other search engines or regions, providing valuable insights for future research. Second, early warning signs must be studied in addition to prediction. Third, we did not test all the methods to make a comparison. Fourth, other factors, such as news reports, may affect search engine search results. Future studies should explore mechanisms to maintain the accuracy of big data prediction. In light of these findings, it is crucial to recognize the broader public health implications of this study. Improving influenza prediction models have far-reaching benefits, including better preparedness and response measures to combat outbreaks. By detecting influenza outbreaks up to 10 days in advance using Baidu search data, we can mobilize health care resources, implement preventive measures, and initiate timely vaccination campaigns. This risk assessment and prediction analysis can also help control emerging respiratory infectious diseases, allowing for prompt interventions to mitigate the scale and impact of epidemics. This approach provides a valuable framework for understanding and managing public health crises, not only in China but globally. By addressing the identified limitations and leveraging the strengths of big data, we can continue to refine predictive models and strengthen global public health preparedness. Early detection and response to infectious disease outbreaks are essential in safeguarding population health, and this study contributes to achieving this critical goal.

In conclusion, this study underscores the potential of combining big data and search engine–based surveillance for influenza prediction, offering insights to revolutionize public health preparedness. By harnessing the power of Baidu search data and others and integrating climate variables, we can enhance early detection and response efforts, ultimately protecting communities from the devastating impact of infectious diseases. As we further explore and optimize these surveillance methods, we envision a future where early warning systems enable swift and effective responses to safeguard global public health.

## Appendix

Multimedia Appendix 1 Supplementary information.

## Abbreviations

DLNM: distributed lag nonlinear model
GFT: Google Flu Trend
PCC: Pearson correlation coefficient

## Data Availability

The influenza virological surveillance data in southern and northern China used in this study are publicly available.
