# Supplementary material for: Influenza Epidemic Trend Surveillance and Prediction Based on Search Engine Data: Deep Learning Model Study
Source: J Med Internet Res. 2023 Oct 17;25:e45085. doi: 10.2196/45085 (PMC10618884; doi:10.2196/45085)
Supplement: Multimedia Appendix 1 [file jmir_v25i1e45085_app1.docx]

**Influenza epidemic trend surveillance and prediction based on search engine data: a deep learning model study**

Supplementary Information

1. Baidu search terms selection

**Table S1.** Baidu search terms selection procedure list.

| Variable | Chinese name | English name | Include(√)  /Exclude(×) | Variable | Chinese name | English name | Include(√)  /Exclude(×) |
| --- | --- | --- | --- | --- | --- | --- | --- |
| Final terms included in this study (VV1-VV37) | | | | | | | |
| VV1 | 病毒性流感 | Viral influenza | √ | VV2 | 季节性流感 | Seasonal influenza | √ |
| VV3 | 流感病毒 | Influenza virus | √ | VV4 | 流感传播途径 | Influenza transmission route | √ |
| VV5 | 流感的传播途径 | How influenza is transmitted | √ | VV6 | 预防流感知识 | Influenza prevention knowledge | √ |
| VV7 | 嗓子痛 | Sorethroat | √ | VV8 | 流感的症状 | Symptoms of the influenza | √ |
| VV9 | 打喷嚏 | Sneeze | √ | VV10 | 乏力 | Lacking in strength | √ |
| VV11 | 肌肉酸痛 | Muscle soreness | √ | VV14 | 感冒清热 | Ganmaoqingre | √ |
| VV13 | 流感丸 | Liuganwan | √ | VV16 | 白加黑 | Baijiahei | √ |
| VV15 | 板蓝根 | Banlangen | √ | VV18 | 达菲 | Tamiflu | √ |
| VV17 | 奥司他韦 | Oseltamivir | √ | VV20 | 流感的预防措施 | Precautions against influenza | √ |
| VV19 | 流感的预防 | Prevention of influenza | √ | VV22 | 怎样预防流感 | How to prevent the influenza | √ |
| VV21 | 预防流感 | Prevent influenza | √ | VV24 | 流感疫苗有必要打吗 | Is it necessary to get a flu vaccine | √ |
| VV23 | 流感疫苗副作用 | Flu vaccine side effects | √ | VV26 | 感冒 | Cold | √ |
| VV25 | 流感 | Influenza | √ | VV28 | 发烧 | Febrile | √ |
| VV27 | 发热 | Fever | √ | VV30 | 咽喉痛 | Pharyngalgia | √ |
| VV29 | 咳嗽 | Cough | √ | VV32 | 肺炎 | Pneumonia | √ |
| VV31 | 流涕 | Runny nose | √ | VV34 | 流感治疗 | Flu treatment | √ |
| VV33 | 胸闷 | Chest tightness | √ | VV36 | 退烧药 | Antipyretic | √ |
| VV35 | 感冒药 | Cold medicine | √ | VV38 | 流感疫苗 | Influenza vaccine | √ |
| VV37 | 连花清瘟 | Lianhuaqingwen | √ |  |  |  |  |
| Inrelevent meaning with influenza (A1-A15) | | | | | | | |
| A1 | 猪流感 | Swine flu | × | A2 | 猪流感病毒 | Swine flu virus | × |
| A3 | 猪流感症状 | Swine flu symptoms | × | A4 | 猪流感的症状有哪些 | What are the symptoms of swine flu | × |
| A5 | 上流感 | Shangliugan | × | A6 | 盲流感 | Blind flu | × |
| A7 | 流感下的北京中年 | Beijing middle-aged under the flu | × | A8 | 流感下的北京中年 原文 | Beijing middle-aged under the flu original text | × |
| A9 | 情流感 | Love flu | × | A10 | 情流感菌 | Love flu bacteria | × |
| A11 | 三月情流感 | March love influenza | × | A12 | 韩国灾难电影 | Korean disaster movie | × |
| A13 | 绿猴 | Green monkey | × | A14 | 看视频花屏 | The screen is not clear when watching the video | × |
| A15 | 什么是禽流感 | What is avian influenza | × |  |  |  |  |
| Not included in the Baidu index database (B1-B13) | | | | | | | |
| B1 | 有一个人患了流感 | One person had the flu | × | B2 | 预防流感手抄报 | Handwritten reports for influenza prevention | × |
| B3 | 流感韩国电影 | Flu korean movie | × | B4 | 尸流感中国 | Corpse flu china | × |
| B5 | 预防流感小常识 | Tips for preventing the flu | × | B6 | 流感疫苗说明书 | Flu vaccine leaflet | × |
| B7 | b型流感嗜血杆菌疫苗 | Haemophilus influenzae type b vaccine | × | B8 | 广州流感 | Guangzhou flu | × |
| B9 | 流感疫苗有必要打 | It is necessary to take the flu vaccine | × | B10 | h3n2甲型流感 | H3N2 influenza a | × |
| B11 | 宝宝流感 | Baby flu | × | B12 | 电影流感 | Influenza movie | × |
| B13 | 流感吃什么好 | What is good to eat for the flu | × |  |  |  |  |
| No search records within one year (C1-C14) | | | | | | | |
| C1 | 鼻喷流感疫苗 | Nasal spray flu vaccine | × | C2 | 电影流感 | Flu movie | × |
| C3 | 韩国电影流感 | Korean movie flu | × | C4 | 流感韩国灾难电影 | Flu korean disaster movie | × |
| C5 | 流感疫苗的最佳接种时间为每年的 | What is the best time to get the flu vaccine every year | × | C6 | 韩国72人接种流感疫苗后死亡 | 72 people died in south korea after receiving flu vaccines | × |
| C7 | 流感疫苗多少钱 | How much is the flu vaccine | × | C8 | 流感疫苗多少钱一针 | How much is a flu vaccine | × |
| C9 | 流感疫苗预约 | Flu vaccine appointments | × | C10 | 美国流感疫情严重 | The flu epidemic in the united states is serious | × |
| C11 | 三价流感疫苗 | Trivalent influenza vaccine | × | C12 | 四价流感疫苗 | Quadrivalent influenza vaccine | × |
| C13 | 西班牙大流感 | Spanish flu | × | C14 | 2022最近爆发的流感叫什么 | What is the name of the recent outbreak of influenza in 2022 | × |
| Subject to geographical interference (D1-D11) | | | | | | | |
| D1 | 北京流感 | Beijing flu | × | D2 | 深圳流感 | Shenzhen flu | × |
| D3 | 香港流感 | Hong Kong flu | × | D4 | 美国 流感 | United States influenza | × |
| D5 | 上海流感 | Shanghai flu | × | D6 | 流感 韩国 | Flu, Korea | × |
| D7 | 韩国电影 流感 | Korean movie flu | × | D8 | 甲流 北京 | Influenza A Beijing | × |
| D9 | 美国流感 | American flu | × | D10 | 欧洲流感 | European influenza | × |
| D11 | 西班牙流感 | Spanish flu | × |  |  |  |  |
| Refers to specified virus strains (E1-E19) | | | | | | | |
| E1 | a型流感 | Influenza A | × | E2 | h1n1流感 | H1N1 influenza | × |
| E3 | 儿童甲型流感症状 | Influenza A symptoms in children | × | E4 | 副流感病毒 | Parainfluenza virus | × |
| E5 | 甲流感 | Influenza A | × | E6 | 甲流感症状 | Influenza A symptoms | × |
| E7 | 甲型h1n1流感 | Influenza A(H1N1) | × | E8 | 甲型h1n1流感病毒 | Influenza A(H1N1) virus | × |
| E9 | 甲型h1n1流感疫苗 | Influenza A(H1N1) vaccine | × | E10 | 甲型流感 | Influenza A | × |
| E11 | 甲型流感病毒 | Influenza A virus | × | E12 | 甲型流感的症状 | Symptoms of influenza A | × |
| E13 | 甲型流感第几天最严重 | What days is influenza A the most serious? | × | E14 | 甲型流感症状 | Influenza A symptoms | × |
| E15 | 流感嗜血杆菌 | Haemophilus influenzae | × | E16 | 新流感 | New flu | × |
| E17 | 新型流感 | Novel influenza | × | E2 | 乙型流感 | Influenza B | × |
| E19 | 致命感冒 | Fatal cold | × |  |  |  |  |
| High correlation with COVID-19 (F1-F7) | | | | | | | |
| F1 | 流感会自己好吗 | Will the flu get better on its own | × | F2 | 流感能自愈吗 | Can the flu heal itself? | × |
| F3 | 流感大流行 | Influenza pandemic | × | F4 | 流感疫苗有必要接种吗 | Is it necessary to get a flu shot? | × |
| F5 | 怎么判断是不是流感 | How to tell if it's the flu | × | F6 | 流感和普通感冒的区别 | The difference between the flu and the common cold | × |
| F7 | 流感发烧一般几天能好 | How many days does flu fever usually get better? | × |  |  |  |  |
| Terms that experts recommend excluding (G1-G9) | | | | | | | |
| G1 | 流感 电影 | Flu; movie | × | G2 | 流感电影 | Flu movie | × |
| G3 | 流感吃什么药 | What medicine to take for the flu | × | G4 | 流感症状 | Flu symptoms | × |
| G5 | 流感疫苗价格 | Flu vaccine price | × | G6 | 流感疫情 | Influenza epidemic | × |
| G7 | 流感预防 | Influenza prevention | × | G8 | 流感最新消息 | Flu update | × |
| G9 | 如何预防流感 | How to prevent the flu | × | G10 | 流感吃什么药效果最好 | What kind of medicine has the best effect on influenza |  |

2. Correlation analysis


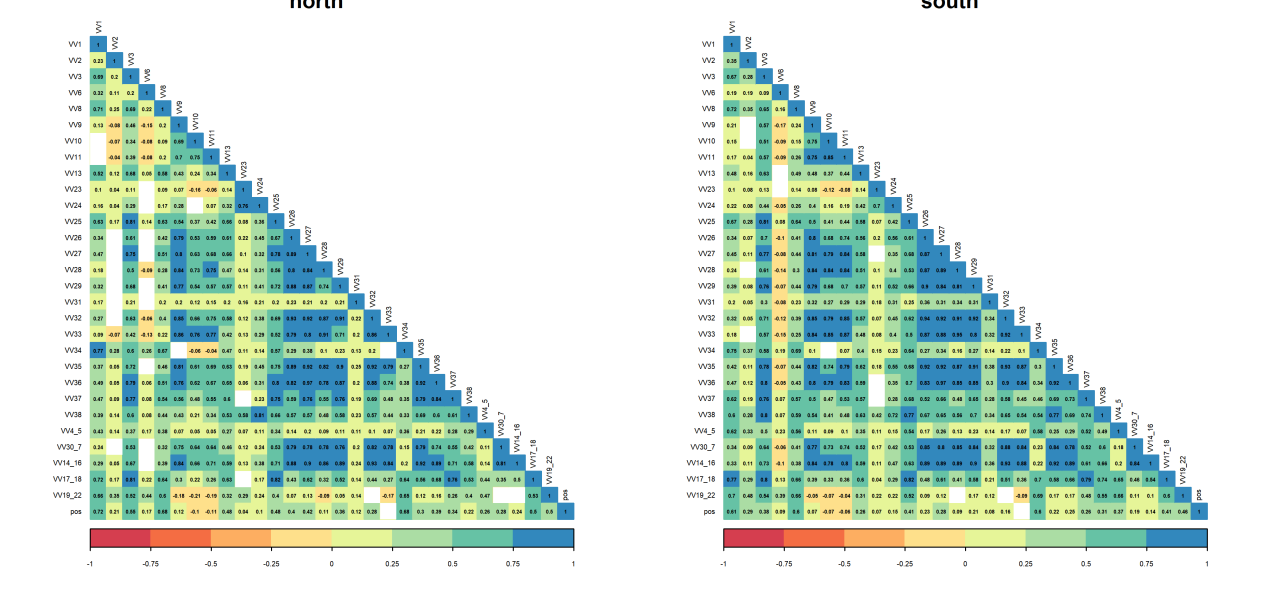


**Figure S1. Bivariate associations between Baidu search terms and influenza positive rate.** **a**. Northern China. **b**. Southern China. Twenty-nine search terms from January 1, 2011, to July 31, 2018, are analyzed by Pearson analysis. Correlation coefficients (PCCs) and the statistical test P-value are provided as lower and upper positions of each penal. Blank refers to *P≥0.05*.

1. **Model parameters**

**Table S2.** GAM model parameters**.**

| Layer (type) | Output Shape | Parameters | Connected to |
| --- | --- | --- | --- |
| input (InputLayer) | (None, 1, 38) | 0 | - |
| gru (GRU) | (None, 1, 512) | 847872 | input |
| gvp1 (GlobalAveragePooling1D) | (None, 512) | 0 | gru |
| gmp (GlobalMaxPooling1D) | (None, 512) | 0 | gru |
| gvp2 (GlobalAveragePooling1D) | (None, 512) | 0 | gru |
| reshape1 (Reshape) | (None, 1, 1, 512) | 0 | gvp1 |
| reshape2 (Reshape) | (None, 1, 1, 512) | 0 | gmp |
| reshape (Reshape) | (None, 1, 1, 512) | 0 | gvp2 |
| cas1 (Dense) | (None, 1, 1, 64) | 32768 | reshape1 reshape2 |
| lambda (Lambda) | (None, 1, 1) | 0 | gru |
| lambda1 (Lambda) | (None, 1, 1) | 0 | gru |
| sb1 (Dense) | (None, 1, 1, 32) | 16384 | reshape |
| cas2 (Dense) | (None, 1, 1, 512) | 32768 | cas1 |
| concatenate (Concatenate) | (None, 1, 2) | 0 | lambda lambda1 |
| sb2 (Dense) | (None, 1, 1, 512) | 16384 | sb1 |
| add (Add) | (None, 1, 1, 512) | 0 | cas2 |
| sa (Conv1D) | (None, 1, 1) | 14 | concatenate |
| sigmoid (Activation) | (None, 1, 1, 512) | 0 | sb2 |
| sigmoid1 (Activation) | (None, 1, 1, 512) | 0 | add |
| sigmoid2 (Activation) | (None, 1, 1) | 0 | sa |
| multiply (Multiply) | (None, 1, 1, 512) | 0 | gru sigmoid |
| multiply1 (Multiply) | (None, 1, 1, 512) | 0 | gru sigmoid1 |
| multiply2 (Multiply) | (None, 1, 512) | 0 | gru sigmoid2 |
| gmp1 (GlobalMaxPooling1D) | (None, 38) | 0 | input |
| flatten (Flatten) | (None, 512) | 0 | multiply |
| flatten1 (Flatten) | (None, 512) | 0 | multiply1 |
| flatten2 (Flatten) | (None, 512) | 0 | multiply2 |
| gmp2 (GlobalMaxPooling1D) | (None, 512) | 0 | gru |
| concatenate1 (Concatenate) | (None, 2086) | 0 | gmp1 gmp2 flatten flatten1 flatten2 |
| dense (Dense) | (None, 30) | 62610 | concatenate1 |

Total params: 1008800

Trainable params:1008800

1. Prediction results

Comparison of prediction effect within different contribution degree of search index


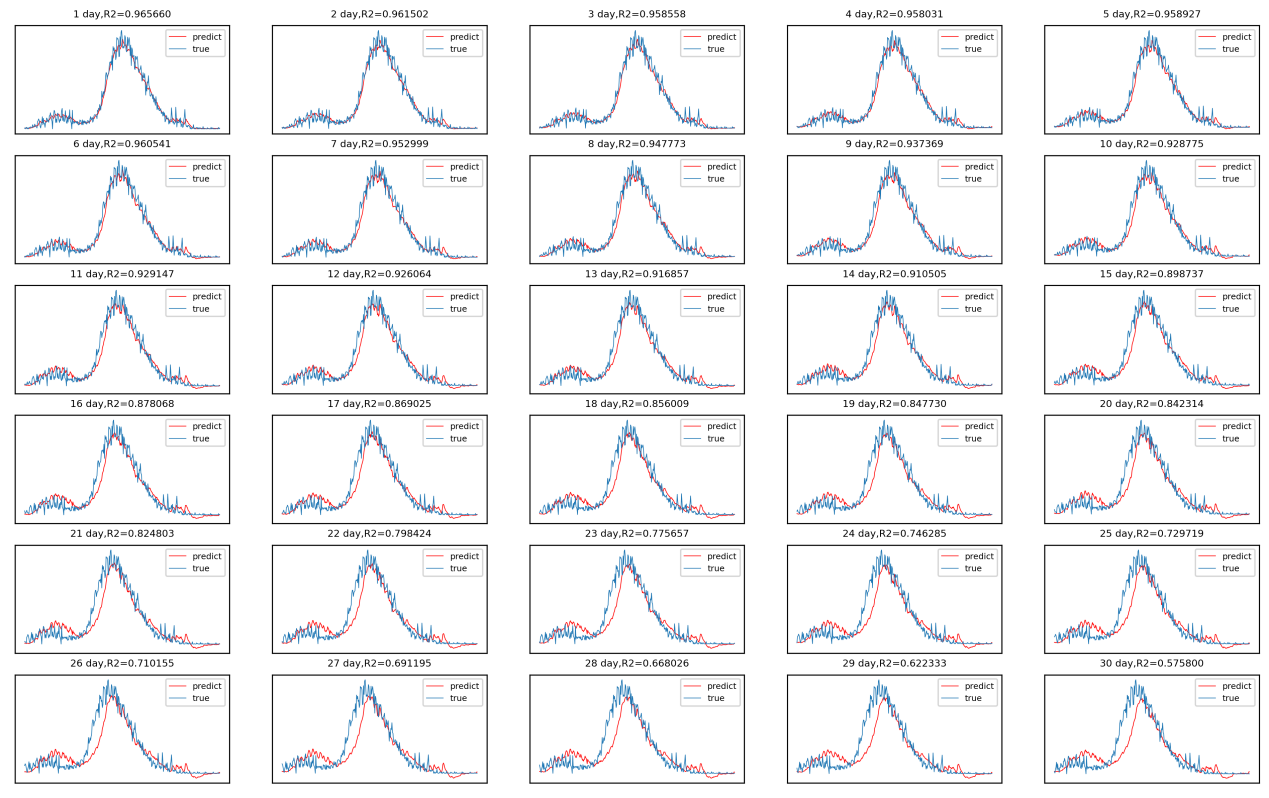


**Figure S2. Prediction of influenza positive rate 1-30 days in advance.** Northern, including the search items whose correlation coefficients p<0.05 with all r rang.


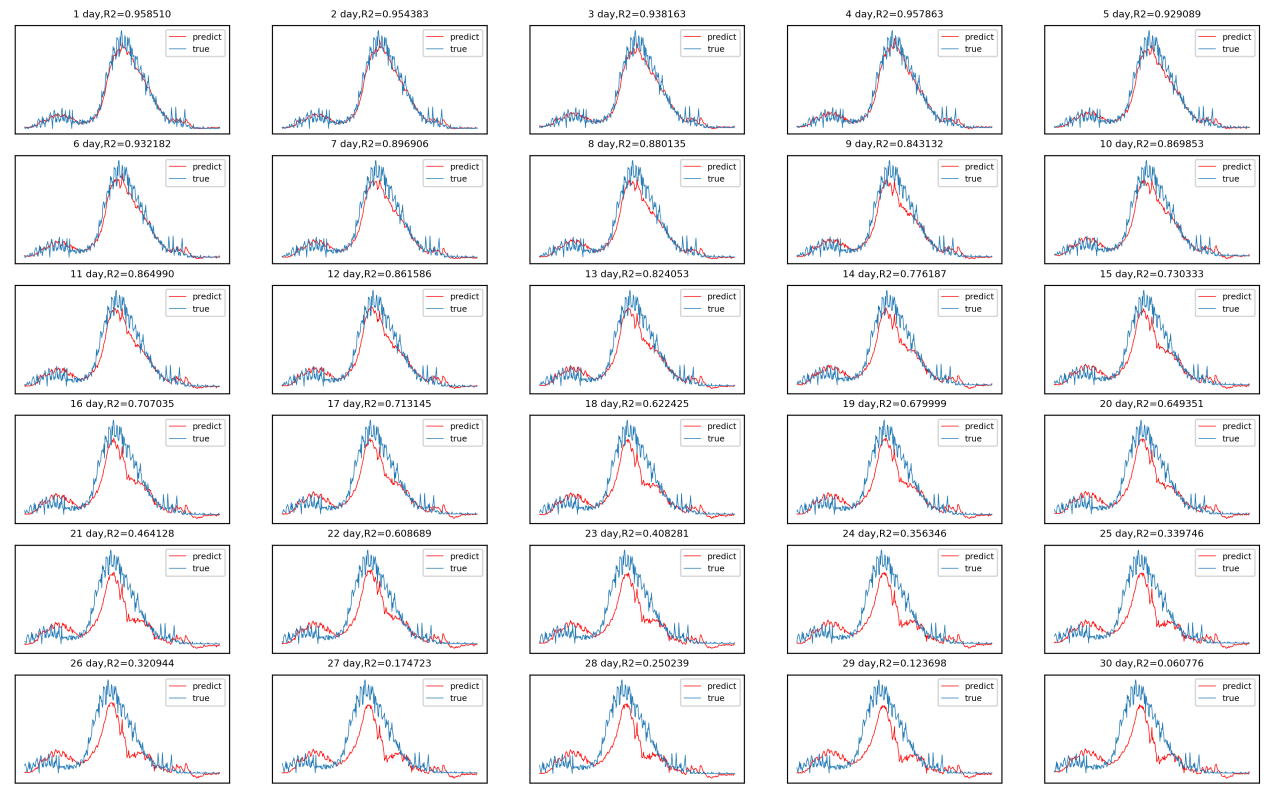


**Figure S3. Prediction of influenza positive rate 1-30 days in advance.** Northern, including the search items whose correlation coefficients p<0.05 with all r≥0.4.


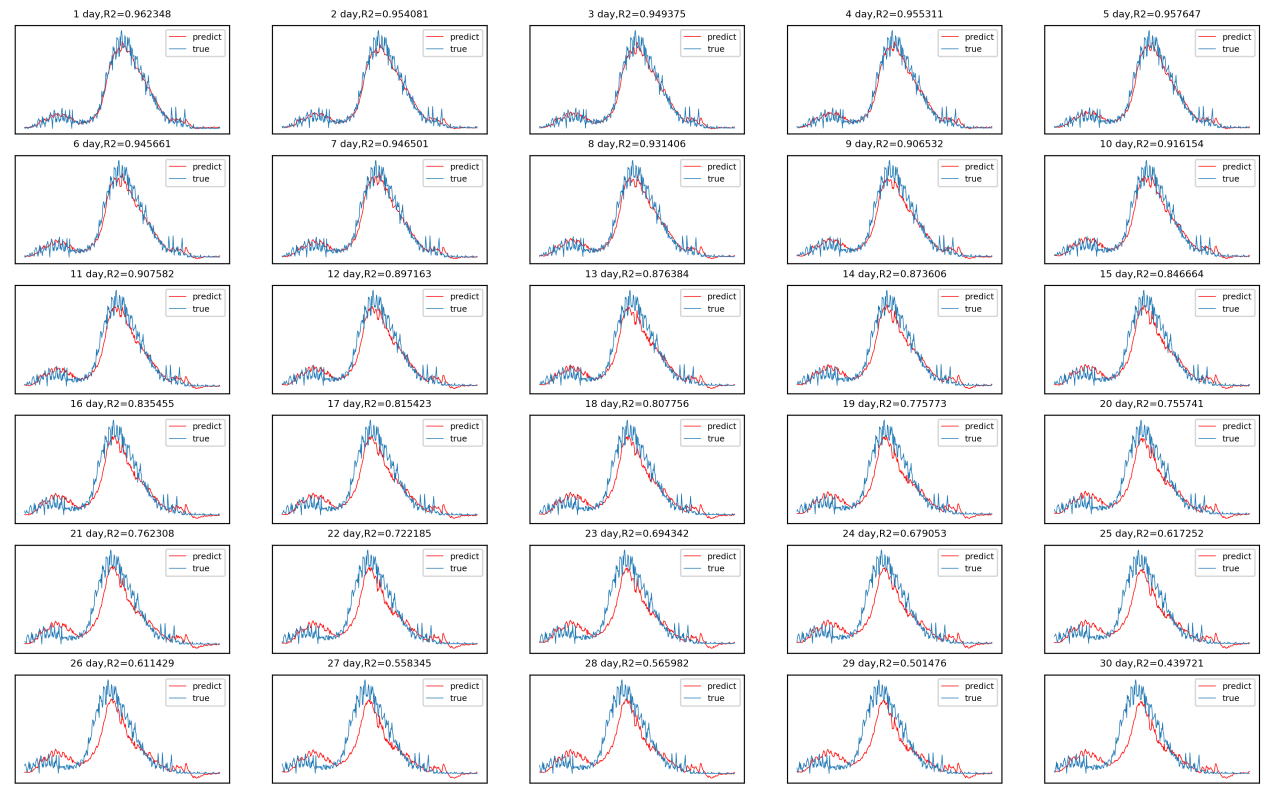


**Figure S4. Prediction of influenza positive rate 1-30 days in advance.** Northern, including the search items whose correlation coefficients p<0.05 with all r≥0.6.


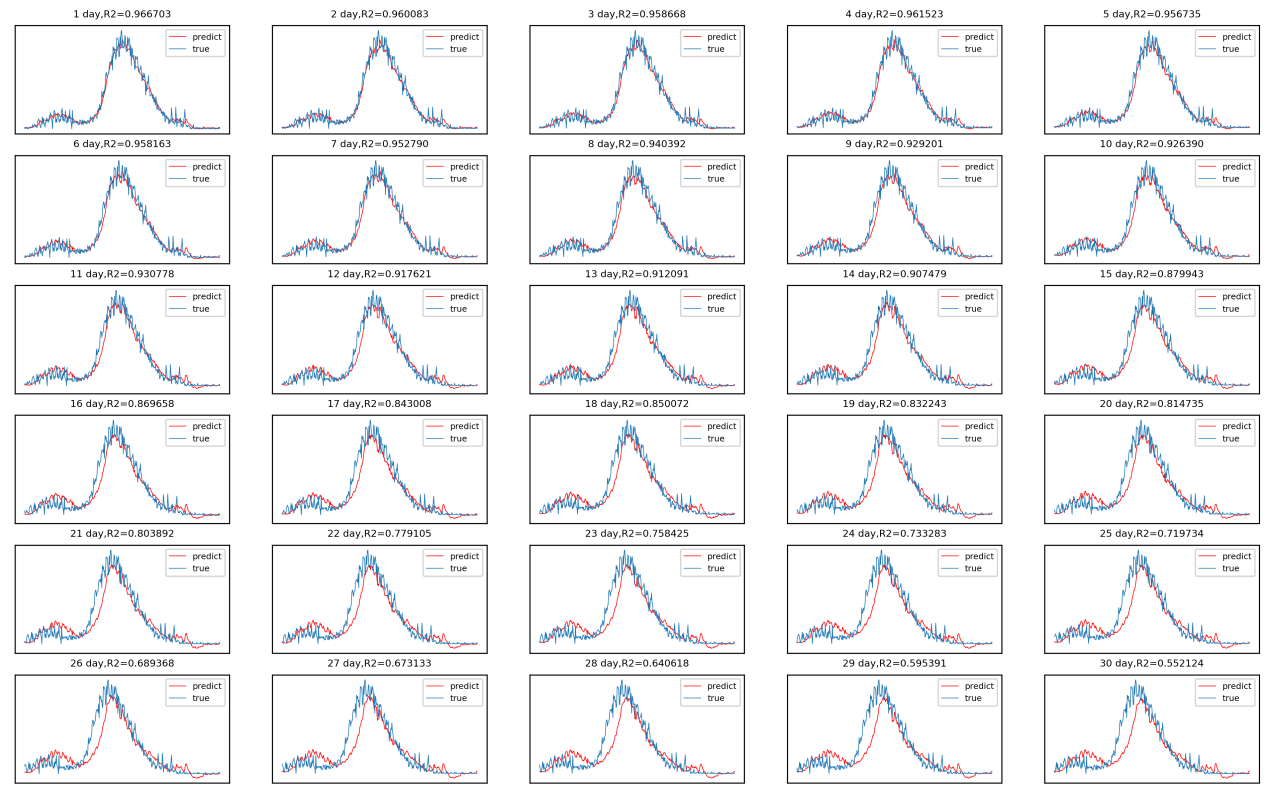


**Figure S5. Prediction of influenza positive rate 1-30 days in advance.** Northern, including the search items whose correlation coefficients p<0.05 with all r≥0.7.


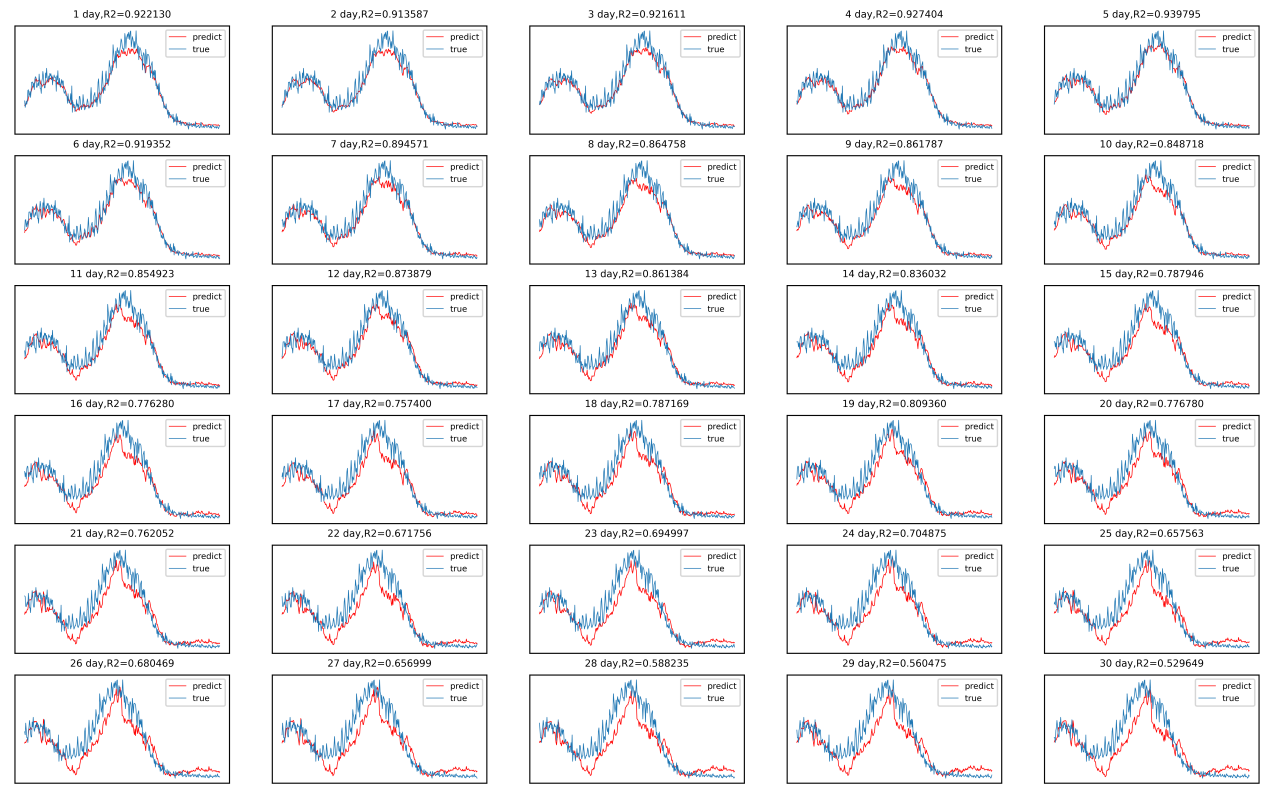


**Figure S6. Prediction of influenza positive rate 1-30 days in advance.** Sorthern, only historical influenza positive rate data with no search items.


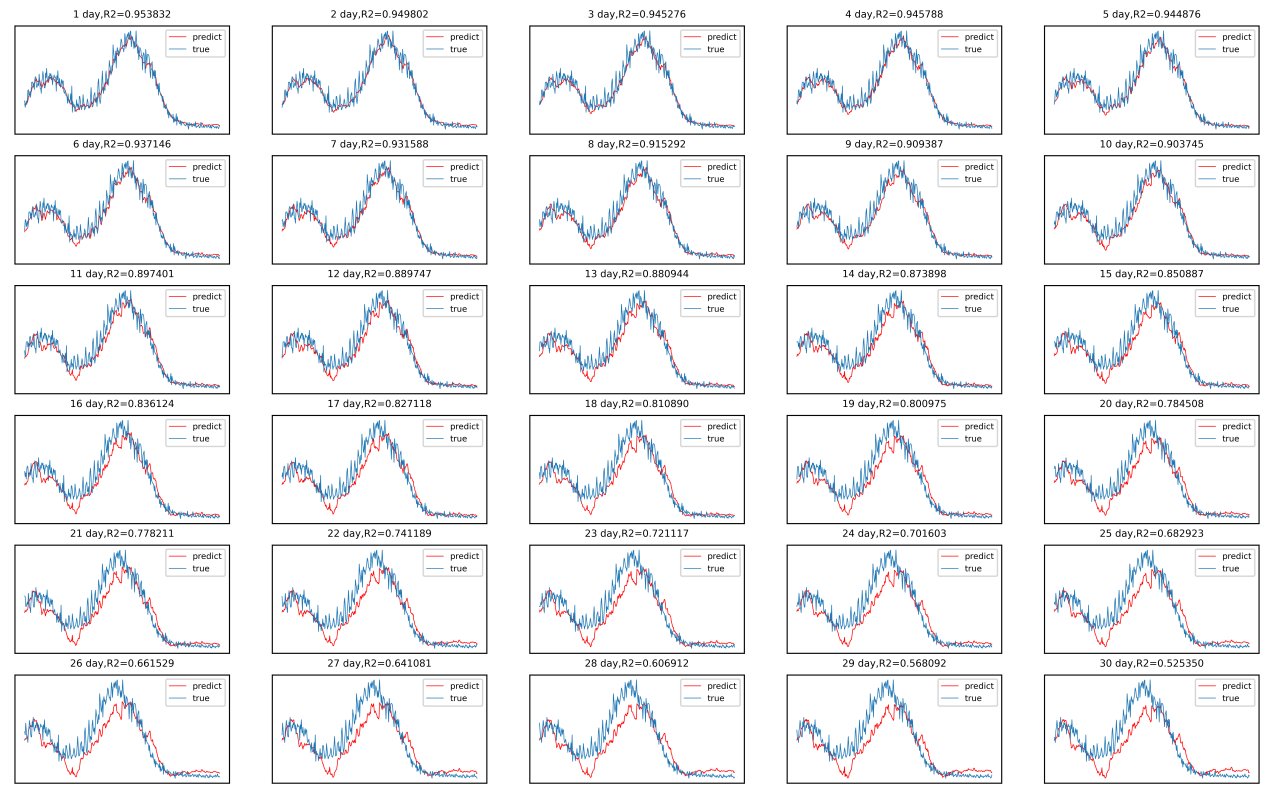


**Figure S7. Prediction of influenza positive rate 1-30 days in advance.** Southern, including the search items whose correlation coefficients p<0.05 with all r ≥0.4.


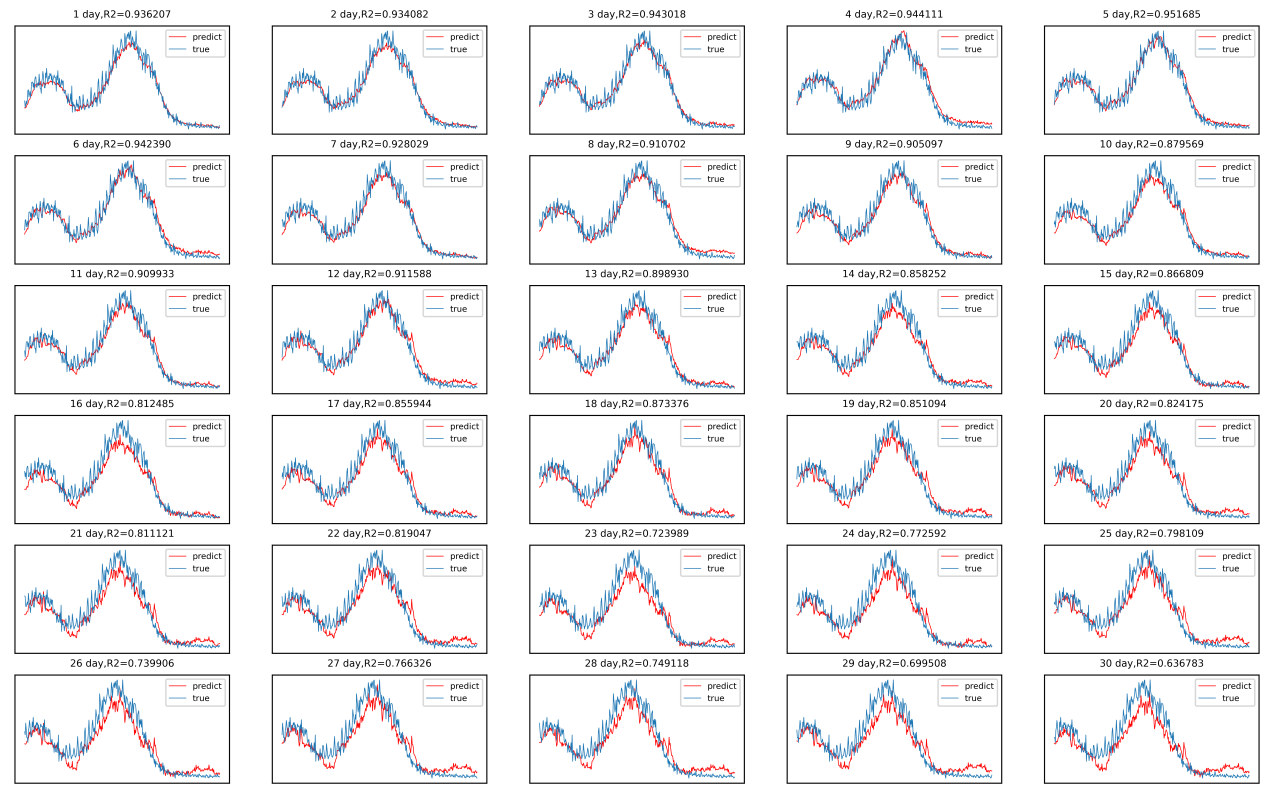


**Figure S8. Prediction of influenza positive rate 1-30 days in advance.** Southern, including the search items whose correlation coefficients p<0.05 with all r≥0.6.
